# Role of EEG as Biomarker in the Early Detection and Classification of Dementia

**DOI:** 10.1155/2014/906038

**Published:** 2014-06-30

**Authors:** Noor Kamal Al-Qazzaz, Sawal Hamid Bin MD. Ali, Siti Anom Ahmad, Kalaivani Chellappan, Md. Shabiul Islam, Javier Escudero

**Affiliations:** ^1^Department of Electrical, Electronic and Systems Engineering, Faculty of Engineering and Built Environment, Universiti Kebangsaan Malaysia (UKM), 43600 Bangi, Selangor, Malaysia; ^2^Department of Biomedical Engineering, Al-Khwarizmi College of Engineering, Baghdad University, Baghdad, Iraq; ^3^Department of Electrical and Electronic Engineering, Faculty of Engineering, Universiti Putra Malaysia (UPM), 43400 Serdang, Selangor, Malaysia; ^4^Institute of Microengineering and Nanoelectronics (IMEN), Universiti Kebangsaan, Malaysia (UKM), 43600 Bangi, Selangor, Malaysia; ^5^Institute for Digital Communications, School of Engineering, The University of Edinburgh, Edinburgh EH9 3JL, UK

## Abstract

The early detection and classification of dementia are important clinical support tasks for medical practitioners in customizing patient treatment programs to better manage the development and progression of these diseases. Efforts are being made to diagnose these neurodegenerative disorders in the early stages. Indeed, early diagnosis helps patients to obtain the maximum treatment benefit before significant mental decline occurs. The use of electroencephalogram as a tool for the detection of changes in brain activities and clinical diagnosis is becoming increasingly popular for its capabilities in quantifying changes in brain degeneration in dementia. This paper reviews the role of electroencephalogram as a biomarker based on signal processing to detect dementia in early stages and classify its severity. The review starts with a discussion of dementia types and cognitive spectrum followed by the presentation of the effective preprocessing denoising to eliminate possible artifacts. It continues with a description of feature extraction by using linear and nonlinear techniques, and it ends with a brief explanation of vast variety of separation techniques to classify EEG signals. This paper also provides an idea from the most popular studies that may help in diagnosing dementia in early stages and classifying through electroencephalogram signal processing and analysis.

## 1. Introduction

Dementia refers to a group of disorders caused by the gradual dysfunction and death of brain cells. This disorder can be described clinically as a syndrome that causes a decline in cognitive domain (i.e., attention, memory, executive function, visual-spatial ability, and language) [1]. Predicting dementia in the early stages would be essential for improving treatment management before brain damage occurs.

The early diagnosis of dementia will help dementia patients start an early treatment based on the symptoms. In the past years, significant advances have been made to reveal the early stages of dementia through biomarkers. These improvements include biochemical, genetic, neuroimaging, and neurophysiological biomarkers [2, 3]. Therefore, developing and integrating these biomarkers to identify dementia in early stages are important to derive an optimal diagnostic index.

In parallel, over the last two decades, significant growth was noted in the research interest on EEG, as the full investigation of neurodynamic time-sensitive biomarker that helps in detecting cortical abnormalities associated with cognitive decline and dementia [4–7]. An EEG marker would be a noninvasive method that may have the sensitivity to detect dementia early and even classify the degree of its severity at a lower cost for mass screening. EEG is also widely available and faster to use than other imaging devices [8, 9].

This review has focused on using EEG as an investigating tool and physiological biomarker to identify dementia in early stages and classify the degree of its severity by signal processing and analysis. The review aims to reveal subtle changes that might define indicators for the early detection of dementia that will help medical doctors and clinicians in planning and providing a more reliable prediction of the course of the disease in addition to the optimal therapeutic program to provide dementia patients additional years of a higher quality of life.

## 2. Dementia and Medical Diagnosis

Dementia occurs when the brain has been affected by a specific disease or condition that causes cognitive impairment [10]. The diagnosis of dementia is usually based on several criteria, such as the medical history of patients with clinical, neurological, and psychological examination, laboratory studies, and neuroimaging [3].

### 2.1. Types of Dementia and Cognitive Spectrum

Dementia is associated with neurodegenerative disorder diversity, as well as neuronal dysfunction and death. Dementia has different types based on its cause; these types include Alzheimer's disease (AD), vascular dementia (VaD), Lewy body, frontotemporal dementia (FTD), and Parkinson's disease, among others [2, 11].

AD and VaD are considered the two most common types of dementia in the world, and thus the present review deals with the effect of AD and VaD on the brain [12]. AD is the most prevalent in the Western world, whereas VaD is the most prevalent in Asia [13].

Half of people aged 85 years or older have AD, and this number will roughly double every 20 years due to the aging population [14, 15]. Several neuropathological changes act together to develop AD. These changes include loss of neuronal cell and development of neurofibrillary tangles and amyloid plaques in the hippocampus, entorhinal cortex, neocortex, and other regions of the brain. These changes can also occur in a nondemented individual, and they are associated with AD development even before typical cognitive symptoms are evident [16, 17]. The reduction in cholinergic tone caused by neural damage results in an increase in cognitive difficulties [18].

VaD is another type of dementia. Between 1% and 4% of people aged 65 years suffer from VaD, and the prevalence for older people doubles every 5 to 10 years [19, 20]. VaD is the loss of cognitive function caused by ischemic, ischemic-hypoxic, or hemorrhagic brain lesions as a result of cerebrovascular disease and cardiovascular pathologic changes, such as ischemic heart disease and stroke [21–23].

**Figure 1:**
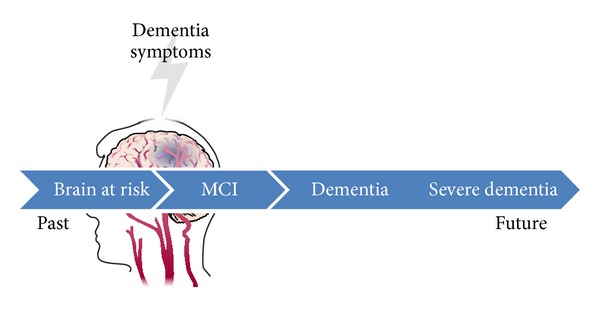
Block diagram of dementia spectrum.

Cognitive impairment introduces individuals to the dementia spectrum that is illustrated in Figure 1. The dementia spectrum can be viewed as a sequence in the cognitive domain that starts from mild cognitive impairment (MCI) and ends with severe dementia, and the period beyond dementia in which the brain is at risk is called cognitive impairment no dementia (CIND) [24].

MCI refers to the decline in cognitive function that is greater than expected with respect to the age and education level of an individual, but the reduced cognitive function does not interfere with daily activities. Clinically, MCI is the transitional stage between early normal cognition and late severe dementia and is considered heterogeneous because some MCI patients develop dementia, whereas others stay as MCI patients for many years. However, patients who were diagnosed with MCI have a high risk to develop dementia, that is, threefold that of people without a cognitive dysfunction. The most commonly observed symptoms of MCI are limited to memory, whereas daily activities of patients remain the same [25].

As dementia diagnosis is not easily performed due to the heterogeneity of the symptoms within the cognitive impairment spectrum, it may be advisable to integrate the neuropsychological testing with biomarkers. The latest diagnosis criteria for AD and MCI support this idea as they highlight the importance that several biomarkers (structural MRI, FDG-PET, and biochemical analyses of the cerebrospinal fluid) have to confirm that a pathological process of AD is, indeed, the cause of the cognitive symptoms [26–29]. The diagnosis criteria usually focus of assessing diverse dementia signs, particularly memory disturbance. The most common diagnosis criteria are developed and characterized by the National Institute of Neurological and Communication Disorder and Stroke-Alzheimer's Disease and Related Disorder Association (NINDS-ADRDA) for AD [26–30] and the National Institute of Neurological Disorders and Stroke and Association Internationale pour la Recherché et l'Enseignement en Neurosciences (NINCDS-AIREN) for VaD [31] and Diagnostic and Statistical Manual of Mental Disorders Fourth Edition (DSM-IV) criteria [32]. The severity of cognitive symptoms could be assessed using Clinical Dementia Rating (CDR) scale [33] and Geriatric Depression Scale (GDS) [34] and Hachinski Ischemic Scale (HIS) [35], whereas the functional outcome can be assessed by instrumental and basic activity of daily living (IDAL) and (BDAL) [36]. The most usable tests to evaluate the early dementia stages even severity of dementia in clinical practice are the Mini-Mental State Examination (MMSE) [37], Montreal Cognitive Assessment (MoCA) [38], and Addenbrooke's Cognitive Examination Revised (ACE-R) [39]. Several validate clinical neuropsychological assessments are used to assess cognitive domain including (but not limited to) Trail Making Test (TMT) [40] and Clock Drawing Test (CDT) [41] for attention and executive function, Rey Osterrieth Figure Copy [42] for construction praxis test, and Phonological and Semantic fluency Token test for language test [43].

### 2.2. Biomarkers for Detecting Dementia

An objective measure, which is related to molecules that are concentrated in the brain or biological fluids or to other anatomical or physiological variables, that help in diagnosing and assessing the progression of the disease or the response to therapies is called a “biomarker.” A biomarker can be used to view the pathogenesis of dementia and helps predict or evaluate the disease risk to identify a clinical diagnosis or therapeutic intervention monitoring that may alter or stop the disease [44, 45]. Ideally, the biomarker should detect the neuropathological processes even before a clinical diagnosis and should help in identifying people who are at risk of developing dementia. The biomarkers for the early detection of dementia may include numerous studies in multiple fields and may be divided into four main categories, namely, biochemical, genetic, neuroimaging, and neurophysiology [2, 3, 11, 46].

#### 2.2.1. Biochemical Marker

Two main types of biochemical markers were identified to reflect the pathological events, particularly detection of dementia, cerebrospinal fluid (CSF), and serum [2, 47].

Several studies have addressed the development in amyloid *β* (A*β*), total tau (T-tau), and hyperphosphorylation tau (P-tau) protein analysis in CSF and plasma as biomarkers for AD. Although CSF biomarkers are specific of AD, Paraskevas et al. [48] has investigate their potential contribution for the differential diagnosis between AD, MCI, and VaD. For instance, A*β*_42_ and T-tau in CSF are useful in differentiating MCI and other dementia stages within the dementia spectrum, whereas the CSF measurement of P-tau and A*β*_42_ can assist in diagnosing VaD or FTD [49]. However, both CSF and serum are used as markers to identify dementia, but the sensitivity and the specificity of these tests are limited [11].

#### 2.2.2. Genetics Biomarkers

Gene expression profile is considered a promising approach for the early detection of dementia. Several studies have been conducted through the genetic analysis of related disorders, such as AD, to evaluate the genetic risk factor that may lead to dementia. Moreover, blood-based gene expression profiling has been described as capable of diagnosing brain disorders by several independent groups. Numerous advantages are offered by the expression profiling of whole blood RNA in deciphering aberrant patterns of gene regulation in neurogeneration. Therefore, the genetic biomarker provides an indication to develop dementia but also needs other biomarkers, such as neuroimaging and chemical biomarkers [2, 3, 11]. The *ε*_4_ allele of the apolipoprotein E gene is the major lipid carrier of protein to the brain, and its inheritance is associated with the onset of AD and VaD. Accordingly, age and the inheritance of the *ε*_4_ allele have been used as a common risk factor and/or pathogenesis for both AD and VaD [45, 50].

#### 2.2.3. Neuroimaging Biomarkers

Neuroimaging has been available for a few decades. This technique can be classified into structural and functional based on the principal information that it provides. Both magnetic resonance imaging (MRI) and computed tomography (CT) are structural imaging techniques; they help clarify the brain diagnosis by detecting the affected area and the type of atrophy or vascular damage. The role of CT is to distinguish two structures and separate them from each other, as CT has good spatial resolution. By contrast, MRI distinguishes the differences between two arbitrarily similar but not identical tissues. MRI provides a good contrast resolution. Positron emission tomography and single photon emission computed tomography are considered functional imaging techniques that can measure brain metabolism parameters, such as regional cerebral blood flow and regional cerebral glucose metabolism. These parameters provide good indication for AD and VaD before morphological changes occur. Moreover, functional MRI is used to measure the brain function over time based on blood oxygen level at rest. It indirectly reflects neuronal activity and identifies the brain activities that are associated with cognitive tasks. Functional imaging techniques are suitable in early dementia detection and diagnosis [2, 3, 11]. These techniques have high spatial resolution for anatomical details but limited temporal resolution. Thus, these neuroimaging techniques are incapable of differentiating the stages within the brain distribution network in series or in parallel activation [51]. Additionally, CT and MRI may be affected by fluid imbibition after brain injury in some cases, thus becoming incapable of detecting the best risk changes or becoming inadequately sensitive to detect dementia in its early stages [52].

#### 2.2.4. Neurophysiological Biomarkers

Neural changes associated with dementia can also be detected with clinical biomarkers, such as EEG, quantitative electroencephalography, event related potential, transcranial magnetic stimulation, and Vagus nerve stimulation [2, 18]. EEG is a neurosignal that tracks information processing with milliseconds precision. It has been subjected to interpretation by clinician visual inspection that results in acceptable and successful diagnosis results. However, EEGs are characterized by spatial resolution that is lower than that of other neuroimaging techniques, although these techniques do not provide functional information about the brain in addition to their limitation in temporal resolution; EEG provides high temporal resolution and it is thus crucial for studying brain activity [53, 54]. Thus, the interpretation of the degree of EEG abnormality and severity of dementia are the benefits of signal processing and analysis of EEG. EEG signal analysis provides a relatively precise localization of electrical activity sources by tracking the hierarchical connectivity of neurons in the recording place. EEG may provide useful indication of the patterns of brain activity if it is integrated with other biomarkers, such as structural and functional neuroimaging [51].

With the dramatic progress in EEG devices, sensors, and electrodes, this review has been focused solely on the function of EEG as a subtle and suitable biomarker in explicitly identifying the neuronal dynamics and cognitive manifestation in most dementia cases, such as AD and VaD, through techniques of EEG signal analysis and processing.

## 3. Function of EEG in the Early Detection and Classification of Dementia

As a neurophysiological biomarker, EEG can characterize different physiological and pathological conditions, such as dementia effects on cortical function distribution. EEG could be used not only as a clinical diagnosis tool, but also as a tool for predicting the stages of dementia [7]. Numerous studies have been conducted to deal with EEG changes associated with dementia and to identify the degree of severity of dementia, and some studies support the possibility for EEG to detect dementia in early stages [55–59]. For instance, Henderson et al. identified dementia presence early through EEG with high sensitivity and specificity [55, 60]; they showed the possibility of using EEG as a marker for AD [61]. EEG may play an important role in detecting and classifying dementia because of its significant influence on dementia abnormalities in terms of rhythm activity. EEG is useful for clinical evaluation because of its ease of use, noninvasiveness, and capability to differentiate types and severity of dementia at a cost lower than that of other neuroimaging techniques [8, 9].

### 3.1. EEG Signal and Mental States

**Figure 2:**
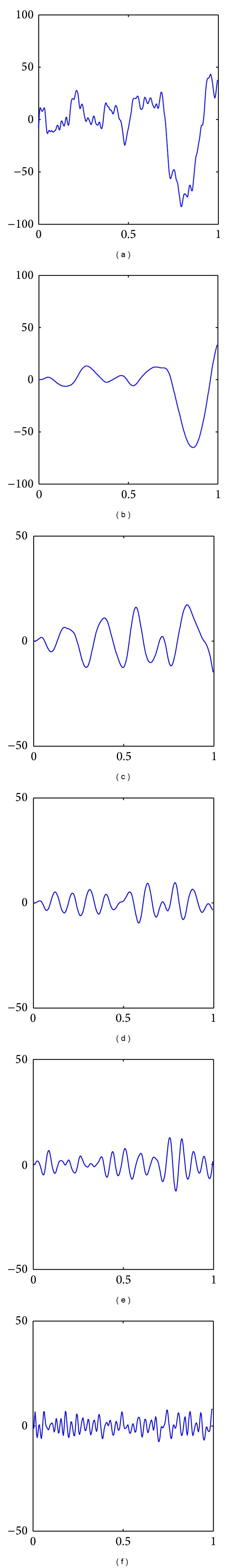
EEG frequency waveform. (a) One second of EEG signal. (b) Delta wave. (c) Theta wave. (d) Alpha wave. (e) Beta wave. (f) Gamma wave.

To deal with EEG signals and to extract useful information and features that help in early dementia diagnosis, an EEG signal should be illustrated in terms of its rhythmic activity [9]. A clinical EEG wave forms an amplitude that is typically between 10 and 100 *μ*v and at a frequency range of 1 Hz to 100 Hz. EEG can be classified into the following five rhythms according to their frequency bands as shown in Figure 2.

1. Alpha (*α*) wave: this rhythmic wave appears in healthy adults while they are awake, relaxed, and their eyes are closed. It occurs at a frequency range of 8 Hz to 13 Hz with a normal voltage range of approximately 20 *μ*v to 200 *μ*v [62]. *α* waveform is diminished by opening the eye, sudden stimulus and attention, and a phenomenon known as alpha blockage or desynchronization. As *α* wave distribution and outcome are based on etiology, the EEG patterns which predominate in the *α* frequency band in case of unconscious or comatose state are defined as alpha coma [63]. *α* rhythm is composed of subunits including alpha1, alpha2, and alpha3, whose spectral band power gives an indication on dementia severity [64, 65]. *α* waveform is mostly observed in the posterior region of the head [66].
2. Beta (*β*) wave: the frequency of *β* waves ranges from 13 Hz to 30 Hz, which is higher than that of the *α* waveform, but their amplitudes are lower and range from 5 *μ*v to 10 *μ*v [62]. *β* waves appear with extra excitation of the central nervous system, increase with attention and vigilance, and replace *α* wave during cognitive impairment. *β* waves are observed in the parietal and frontal region of the scalp [66].
3. Theta (*θ*) wave: the frequency range of *θ* wave is 4 Hz to 7 Hz. This waveform is prominent during sleep, arousal in older children and adults, emotional stress, and idling. *θ* wave is recorded across the temporal and parietal region of the scalp with an amplitude range of 5 *μ*v to 10 *μ*v [62]. Two types of *θ* are found among adults based on their activity; the first type shows a widespread distribution across the scalp and is associated with decreased alertness, drowsiness, cognitive impairment, and dementia, whereas the second type is called frontal midline theta because it is distributed within the frontal midline and is generated by the anterior cingulated cortex, which is the largest region with a positive correlation between the theta current density and glucose metabolism. This wave has been linked to activities such as focusing, attention, mental effort, and stimulation processing [66].
4. Delta (*δ*) wave: the lowest frequency of *δ* wave is less than 3.5 Hz, and its amplitude ranges from 20 *μ*v to 200 *μ*v. *δ* wave occurs during deep sleep, in infancy, and with serious organic brain diseases. This waveform can be recorded frontally in adults and posteriorly in children [62].
5. Gamma (*γ*) wave: the frequency of *γ* wave ranges from 30 Hz to 100 Hz [62]. This waveform is recorded in the somatosensory cortex in the case of cross model sensory processing, during short-term memory to recognize objects, sounds, tactile sensation, and in pathological case because of cognitive decline, particularly when it is related to *θ* band [66].

Until late adulthood, the activities of *δ* and *θ* waves diminish with age, whereas those of *α* and *β* waves increase linearly [67]. The current density of *δ* and glucose metabolism possess an inverse relationship in the case of cerebrovascular diseases, such as stroke, and may be found within the subgenual prefrontal cortex as an outcome of dementia cognitive impairment [68].

### 3.2. EEG Finding in Dementia

EEG has been used as a benchmark for the detection and diagnosis of dementia for two decades. Numerous studies have supported the capability of EEG recording to detect AD and VaD early [59, 69]. Other studies have used EEG as a tool for differentiating AD from other types of dementia, particularly in the differential diagnosis of AD and VaD [70, 71]. EEG can diagnose the two most common types of dementia (i.e., AD and VaD) because both of these types are cortical, and EEG reflects hidden brain abnormalities [72, 73].

The first EEG clinical observation was illustrated by Berger in the beginning of the last century [74, 75]. The interpretation of the conventional visual characteristics related to AD can be summarized by slowing the EEG dominant posterior rhythm frequency, increasing the diffused slow frequency, and reducing both alpha and beta activities, whereas the occipital alpha activity is preserved and theta power is increased in the case of VaD. The delta power is increased in both AD and VaD patients [4, 76]. The computerized EEG signal analysis provides quantitative data, including reduced mean frequency, increased delta and theta power along with decreased alpha and beta power, reduced coherence in the cortical area, and reduced EEG complexity in dementia patients [4]. Numerous studies by Moretti et al. investigate subrhythms within alpha, where the power ratio of alpha3/alpha2 is used as an early marker for prognosis of MCI and the increase in this ratio is correlated with hippocampal atrophy in both MCI and AD patients, whereas theta/alpha1 ratio could be as a reliable index for cerebrovascular damage [64, 77–79]. However, EEG may exhibit normal frequency and may appear similar to normal aged control subjects during the earliest stages of dementia [4]. Nonetheless, EEG signal analysis may contribute to the deeper understanding of dementia because such computerized analysis provides quantitative data instead of mere visual inspection.

### 3.3. EEG Signal Processing

**Figure 3:**
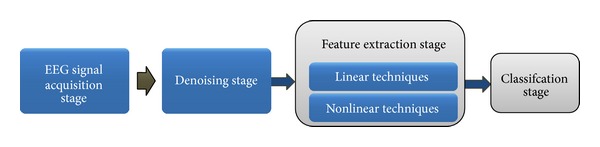
EEG signal processing main stages.

The recorded EEG needs successive stages of signal processing to extract meaningful markers from the EEG signal of dementia patients, and these markers reflect brain pathological changes. The main stages of EEG signal processing are denoising, feature extraction, and classification.  Figure 3 illustrates the stages of EEG signal processing.

#### 3.3.1. EEG Signal Acquisition Stage

EEG is a medical device that reflects the electrical activity of the neurons of the brain and records from the scalp with metal electrode and conductive media [80].

**Figure 4:**
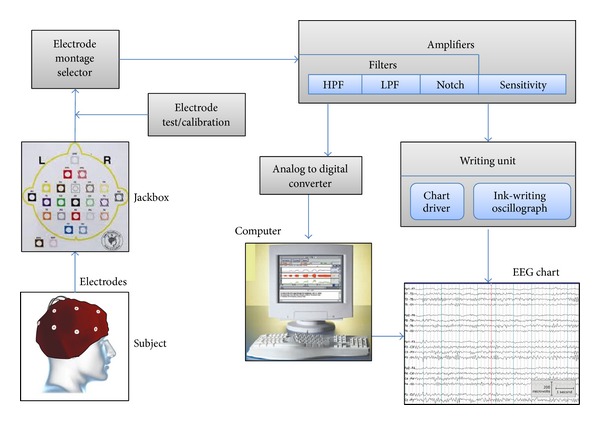
EEG machine schematic diagram.

Figure 4 shows the general EEG machine schematic diagram; it consists of electrodes, amplifiers, A/D converter, recorder, storage, and display devices.

**Figure 5:**
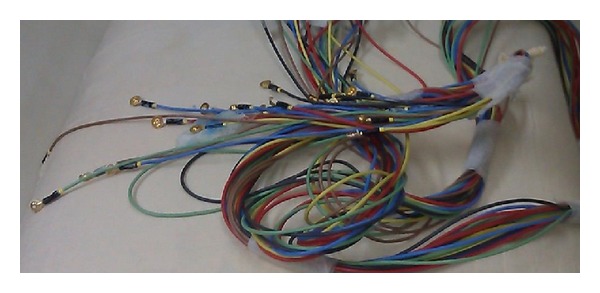
EEG cap electrodes.

For dementia patients, several procedures have been proposed to record the EEG signal; for instance, the gold plate cup electrodes shown in Figure 5 have been used to record EEGs. The skin should be swabbed with alcohol and gel or paste should be applied before placing the electrode on the scalp to reduce the movement of the device and improve the electrode conductivity; the EEG electrode-scalp contact impedance should be below five kilo-ohms to record good quality signal [81].

**Figure 6:**
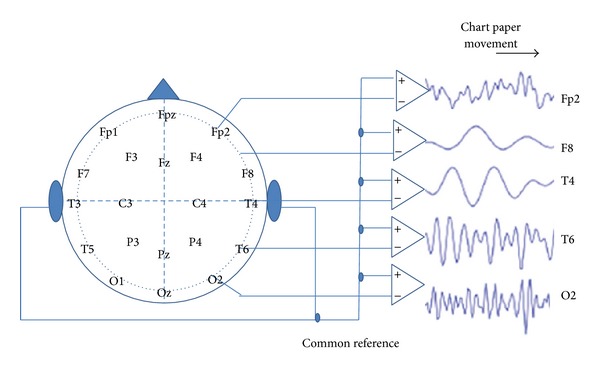
EEG referential montage.

Referential montage is the most popular montage used for EEG recording for dementia that is employed to record the voltage difference between the active electrode on the scalp and the reference electrode on the earlobe, for example, as shown in Figure 6 [82, 83].

**Figure 7:**
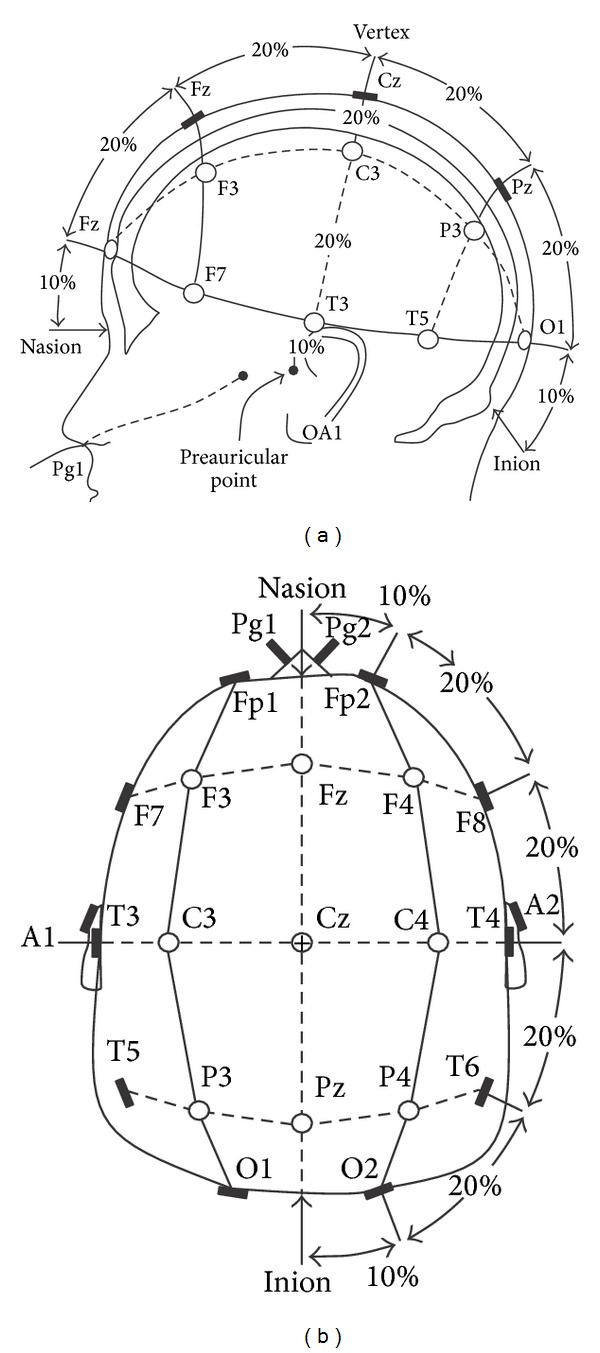
The 10–20 EEG electrodes placement system. (a) and (b) Three-dimensional side view and top view, respectively [85].

For fruitful clinical application, the EEG of dementia patients has been recorded in a specialized clinical unit state with the 10–20 system of the international federation, which is adopted by the American EEG Society, while resting with eyes comfortably closed, as shown in Figure 7. Hamadicharef et al. [84] used 19-recording electrodes plus ground and system reference for EEG recording for dementia patients; these electrodes were located according to 10–20 electrode system as follows: Fp1, Fp2, F7, F3, Fz, F4, F8, A1, T3, C3, Cz, C4, T4, A2, T5, P3, Pz, P4, T6, O1, and O2 [85].

An example of the most popular EEG device contains a low pass, high pass, and notch filters. Typical frequency values for low pass filter (LPF) (i.e., 3 dB) are 0.16, 0.3, 1.6, and 5.3 Hz, and the upper cutoff frequency can be, for example, 15, 30, 70, or 300 Hz. Typically, the frequency for EEG recording for dementia range is from 0.3 Hz to 70 Hz, and the notch filter is 50 Hz or 60 Hz [86]. The sampling frequency can be 128 Hz, 173 Hz, or even higher such as 256 Hz and it is selected based on the application with a 12 bit or 16 bit A/D converter digitalizing the signal to be more accurate. Finally, the EEG signal will be printed on papers, displayed on the computer screen, and stored for further examination in the next stage [81].

#### 3.3.2. Denoising Stage

The reliability of the recorded EEG signal is heavily affected by its noise factors. Most artifacts overlap with the frequencies of EEG signals. The artifacts that contaminated the EEG signal are divided into physiological (e.g., muscle activity, pulse, and eye blinking) [87–90] and nonphysiological artifacts (e.g., power line interference noise and sweat) [90, 91] and/or neuronal activity (e.g., background). The noise has a direct effect on EEG signal properties, and thus different signal processing techniques have been applied to overcome this problem and to extract relevant information from the recorded EEG signal. In order to focus on the role of EEG in the diagnosis of dementia, the mathematical details have been simplified in the text. This section discusses the most popular and effective methods used for EEG denoising.

Independent component analysis (ICA) is a blind source separation higher order statistical method used to split a set of recorded EEG signal (i.e., mixed signals) into its sources without previous information about the nature of the signal. Langlois et al. and McKeown et al. used ICA to observe the EEG signal mixture that reflects multiple cognitive activities or artifacts, particularly the ocular artifact [92–94].

**Figure 8:**
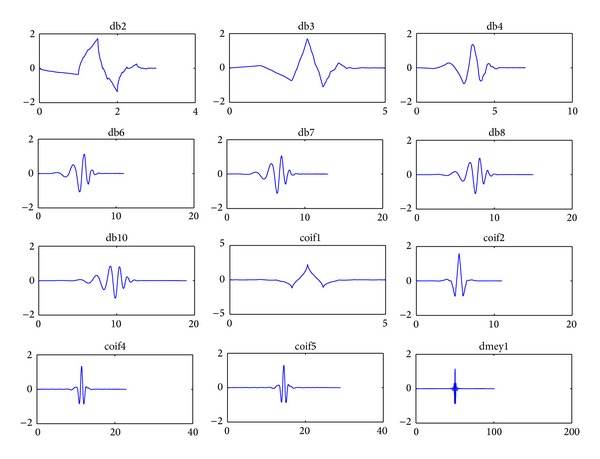
Examples of mother wavelet of Daubechies, coiflets, and dyme.

Wavelet transform (WT) is an effective denoising procedure that was introduced to process nonstationary signals, such as EEG. Zikov et al., Krishnaveni et al., and other researchers used WT to remove ocular artifact [95–98]. The continuous wavelet transform (CWT) can be used as a set of decomposition functions called mother wavelet; the most popular mother wavelets used in biomedical signal denoising are Daubechies, coiflets, and dyme, as shown in Figure 8.

**Figure 9:**
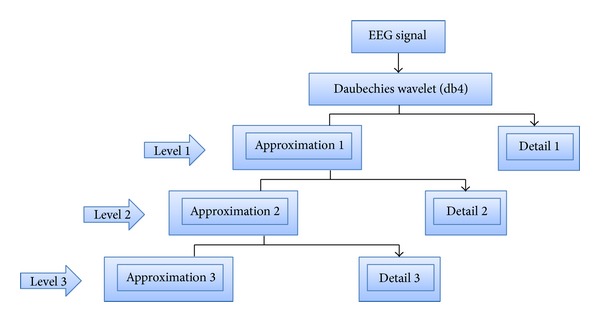
Wavelet multiresolution analysis.

WT is considered a method for multiresolution analysis that provides varying resolutions at different time and frequency [99], as shown in Figure 9.

**Figure 10:**
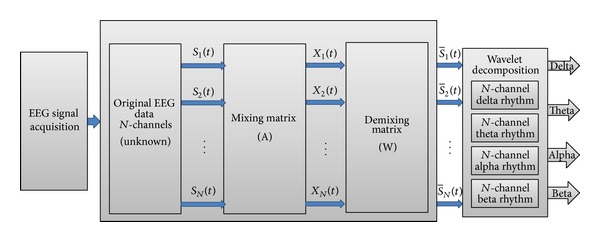
Block diagram of ICA-Wavelet for EEG denoising.

Nazareth et al. and other researchers applied an effective new approach by combining ICA and WT resulting in an ICA-WT hybrid technique, as shown in Figure 10. As an example, ICA-reconstructed data were cascaded as an input to WT decomposition. This merge assists ICA in distinguishing the signal and noise even if both nearly have the same or higher amplitude and removes overlapping noise signal. Furthermore, the WT can decompose EEG signals into different subbands based on the decomposition levels [100–105]. The ICA-WT technique has illustrated successful results in removing the electrooculography and muscle activity artifacts [105]. Accordingly, this technique is useful in revealing hidden EEG characteristics by the next stage. Thus, the signal is ready for the next stage (i.e., feature extraction stage).

#### 3.3.3. Dementia Feature Extraction and Selection

The denoised EEG signal from the previous stage undergoes feature extraction to detect dementia and develop a useful diagnostic index using EEG. This stage aims to extract the useful information from the EEG of dementia patients by linear and nonlinear techniques.

Linear techniques have been used to extract meaningful features from the EEG of dementia patients that are useful as early dementia indices. Jeong used linear techniques based on coherence and spectral calculations that were used to find EEG abnormalities [4]. A slowdown in EEG signals in dementia is illustrated by the shifting of power to the lower frequency and the decrease in interaction among the cortical area (i.e., increase in delta and theta power along with decrease of alpha power) [106]. Spectral analysis has intensively been used to gain insight into dementia, for instance, Escudero et al. [107] have analyzed the magnetoencephalogram (MEG) signals to quantify their abnormalities in the spectra of dementia patients with two spectral features (i.e., the median frequency (MF) and the spectral entropy (SpecEn)) based on their usefulness in distinguishing the brain activity of dementia patients from the normal-age match subjects. Both spectral features provide information about the relative power of low and high frequencies that reflect the local synchronization of the neural assemblies [108]. Thereafter, the electrical brain activity for dementia patients is characterized by the slowing of brain frequency, and this property can be performed using MF and SpecEn [107].

AD and VaD patients share spectral analysis properties, such as the slowing of alpha power and increase in delta power, but theta power is higher in VaD patients than in AD patients [86]. However, EEG frequencies may look normal in the early stages of AD [109]. Generally, the severity of cognitive impairment and the degree of EEG abnormalities are correlated [4].

EEG coherence is used to evaluate the cortical connection functionality and quantify cortico-cortico or cortico-subcortical connection. Moreover, the coherence function can be used to quantify the linear correlation and detect the linear synchronization between two channels; however, this function does not distinguish the directionality of the coupling [110, 111]. A decrease in coherence is interpreted as a reduction in linear function connection and function uncoupling in the cortical area. By contrast, an increase in coherence is interpreted as augmented linear function connection and function coupling in the cortical area [51].

Nonlinear dynamic techniques have been used intensively to analyze the EEG signal, particularly the EEGs of dementia patients, for decades. Researchers have used EEG to investigate the complex dynamic information that is reflected from the brain cortex and recorded by EEG devices [112, 113]. The hypothesis that the brain is stochastic may be rejected based on the capability of the brain to perform sophisticated cognitive tasks thanks to its complicated structure. Moreover, brain neurons are controlled by nonlinear phenomena, such as threshold and saturation processes, such that brain behavior can be classified as nonlinear. The nonlinear dynamic analysis may be considered a complementary approach in detecting mental diseases, because it provides additional information to that of traditional linear methods [114, 115]. Moreover, numerous methods have been introduced to study time series EEG data from human brain activity to understand and detect EEG abnormalities.

The first nonlinear methods that were used to analyze EEG are the correlation dimension (*D*_2_) and the first Lyapunov exponents (*L*_1_).* D*_2_ was applied by Grassberger and Procaccia in 1983 to quantify the number of independent variables that are necessary to describe the dynamic system. It was used to provide the statistical characteristic of the system. By contrast,* L*_1_ was applied by Wolf in 1985 as a dynamic measure to gauge the flexibility of the system [116, 117]. Early detection of dementia can be predicted using fractal dimension (FD), zero-crossing interval (ZCI), entropy, such as sample entropy (SampEn) and Kolmogorov entropy, central tendency measure, and Hojorth-Index. Hamadicharef et al. presented the performance results of these methods based on sensitivity, specificity, accuracy, area under the ROC curve, and standard error and found that FD and ZCI are the best methods [118].

Henderson et al. successfully applied FD as a powerful tool for transient detection in terms of waveform that is used to measure the signal structure details in biology and medicine. The derivation of FD of the autocorrelation function can be found in [119]. Moreover, they used ZCI to analyze EEG [55].

Lempel-Ziv-Welch (LZW) is a metric that has been applied to evaluate the signal complexity by measuring the number of distinct substring and their rate of recurrence along the time series; Ferenets et al. introduced an algorithm to compute the LZW [120].

Several methods have dealt with the complexity or irregularity in the ability of the system to create information by entropy methods, such as Tsallis entropy (TsEn), approximation entropy, SampEn, and multiscale entropy (MSE) [55, 61, 115, 121–124].

To sum up, linear spectral methods have been used traditionally in the field. Their interpretation may be more straightforward for clinicians, as they are closely related to the power associated with different brain rhythms (alpha, beta, delta, and theta), whereas nonlinear techniques may provide complementary information. Nonlinear methods are motivated by the nonlinear behavior of the neurons in the brain. Both approaches have been used to inspect the EEG activity in dementia, but most studies have focused on only one of those families of methods and there are few comprehensive comparative studies [108]. Despite potentially promising findings, the sizes of the analyzed datasets limit the results. These features are applied to the next stage to estimate the degree of the severity of dementia.

#### 3.3.4. Dementia Classification Techniques

**Figure 11:**
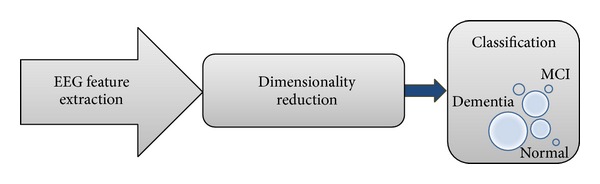
Block diagram of EEG features dimension reduction.

The classification staging is necessary to predict the qualitative properties of the mental state of dementia patients. In this stage, the feature vectors extracted from the previous stage were classified into three categories, namely, CIND, MCI, and dementia. Feature vectors must be analyzed further before being applied to the classifier to avoid overloading the classifier and reduce the computational time, increasing the accuracy of classification. These feature vectors can be processed using dimensionality reduction techniques as shown in Figure 11. Numerous methods can be used including principal component analysis (PCA) and ICA. These methods are well-established methods for dimensionality reduction. PCA is a widely used method to avoid the redundancy because of high-dimensional data [125–127]. The dimensionality-reduced features were used as an input to the classifiers to improve the accuracy of the classification of the severity of dementia by EEG signal analysis.

In EEG applications, highly accurate classification is strongly related to the quality of extracted features, the dimensionality reduction, and the classifiers. Linear discriminant analysis (LDA) and support vector machine (SVM) classifiers are the most popular methods used to classify brain disorders, such as dementia and epilepsy, because of their accuracy and applicability in numerous studies [125, 126].

LDA has been widely used for its fast and simple implementation with low computational requirements. It is suitable for real-time implementation [128]. Its objective is to create a new variable that combines the original predictors by finding a hyperplane that separates the data points representing different classes and that minimizes the variance within the class under the assumption of normal data distribution [125].

**Figure 12:**
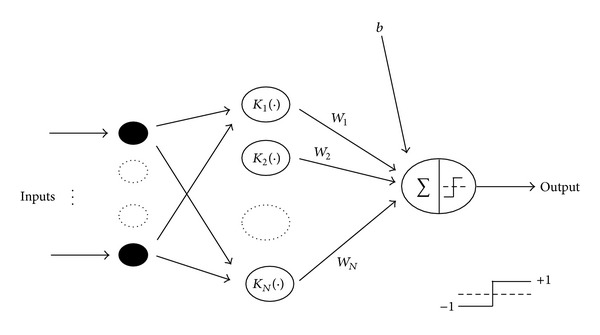
Support vector machine classifier [130].

SVM is a linear binary classifier that can be used as an alternative to multilayer perceptron. It can support feature vectors with many components [129]. SVM gives the researchers a way to come up with a nonlinear classifier by appropriate kernel methods. Specifically, SVM uses hyperplanes that maximize the distance between the two classes of SVMs based on the principle of maximizing the margin of separation of the classifier to split class [130]. In nonlinear cases, SVM can be extended to the concept of hyperplane separation of data that are often linearly nonseparable. The two classes are mapped with kernel methods onto a new higher dimensional feature space via nonlinear mapping [130].  Figure 12 shows the architecture of the SVM.

SVM is widely used in biomedical signal classification application, particularly in EMG and EEG classifications, for its high accuracy and good performance that make it insensitive to overtraining and dimensionality [126, 132–134]. SVM may help obtain an accurate classification of the severity of dementia that provides an indication of the mental disorder and can predict early stages with suitable treatment management programs.

## 4. Discussion

EEG plays an important role in evaluating brain activity. This review is focused on using EEG as a physiological biomarker to detect dementia in the early stages and classifying its severity based on EEG signal analysis and processing.

There is enormous interest in the detection and diagnosis of dementia in its early stages. This might be achieved through a combination of diagnosis criteria and reliable biomarkers. The scientific knowledge available through neuropsychological testing and biomarkers assessed against diverse dementia signs would help in capturing both the earliest stages and the spectrum of dementia before significant mental decline [26–29]. There is an urgent need for an accurate, specific, and cost-effective biomarker to diagnose dementia. This makes the EEG an attractive tool to detect and differentiate AD and VaD in the early stages due to its affordability and noninvasiveness. This review has focused on the use of EEG as a physiological biomarker to provide the impetus to detect dementia in the early stages. EEG evaluation through visual inspection is prone to mistakes due to the subjective experience of neurologists. In addition, it is time consuming and it may not be able to reveal subtle changes in the EEG, whereas the computerized EEG signal analysis may simplify the work of medical doctors and may contribute to making the evaluations more objective.

This review illustrated EEG signal processing principles and described useful techniques that have been used to enhance recorded EEG signals. Numerous preprocessing signal and denoising techniques are used to enhance EEG signals by removing artifacts. Methods like WT and ICA have been used to remove different types of noise. On the one hand, ICA, as a higher order statistics method, has several advantages due to its ability to split a set of mixed signals into its sources. Nonetheless, ICA may have difficulties in determining the order of the ICs. However, it is a powerful method for artifact removal and suitable for offline application. On the other hand, WT is suitable for nonstationary signal like EEG that provide linear combination of the sum of wavelet coefficients and mother wavelet with frequency and localization information, and WT has the ability of splitting the signal into subbands (approximation and detail) using a multiresolution decomposition algorithm. In recent years, ICA-wavelet hybrid techniques have been used to overcome the limitation of each individual method and it may become a more effective denoising method. To improve the performance of ICA and WT, the data can be projected into a new space when the redundancy is higher and the features in frequency domain are fully exploited. This minimizes the information loss and it enables WT to remove any overlapping of noise in the EEG signals that ICA cannot filter out.

This review also explored linear and nonlinear features extraction techniques and dimensionality reduction methods. The summary of the findings of the most effective linear and nonlinear methods is listed in Table 1.

Thereafter, the techniques used to classify EEG signals based on dementia spectrum (i.e., CIND, MCI, and dementia) were revised. The effects of dementia on the EEG can be summarized as slowing and reducing EEG complexity and synchrony. The SVM classifier is suggested as a suitable technique for classifying the features of EEG signals based on their applicability in many fields for its empirically good performance and generalization. Many researchers have benefited from the advantages of SVM in dealing with large feature spaces. Other researchers have been applying a combination of classification algorithms that may help improve the performance, sensitivity, and specificity of the best clinical diagnosis for the early detection and classification of dementia.

## 5. Conclusions 

In this review, EEG has been identified as an investigation tool and potential biomarker for detecting dementia and classifying its severity by providing concise information about the brain activity and how it is affected by AD and VaD. It must be noted that, in some occasions, the review has focused on findings related to AD. This is due to the fact that the literature on AD is much larger. Although there has been considerable research into the use of EEG for dementia screening, this is not accepted in routine practice yet [26–29]. Furthermore, the analyzed datasets have often been small and additional studies are needed to confirm those promising results. However, several studies have appreciated the EEG as a useful clinical evaluation tool in the discrimination of AD and/or VaD and/or other types of dementia. Highly sensitive EEG-based detection of the progress of dementia and classification of its severity are a highly desirable screening technique in clinical practice as its low cost and portable features make it a promising technique that can be a reference for customizing or personalizing optimal therapeutic programs for dementia patients.

## Figures and Tables

**Table 1:** Findings of effective linear and nonlinear methods for detecting dementia [55, 107, 121, 122, 131].

	Methods		Finding
Linear techniques	Spectral analysis	Median frequency (MF)	Dementia is associated with a slowing of brain frequencies
		Spectral entropy (SpecEn)	Dementia causes a change in the frequency content of the brain signals
	Zero crossing interval (ZCI)		ZCI increased in slow activity associated with dementia

Nonlinear techniques	Fractal dimension (FD)		FD of the EEG is lower for dementia patients than normal subjects
	Lempel-Ziv-Welch (LZW)		Lower LZW of dementia patients than normal subjects due to reduce complexity
	Tsallis entropy (TsEn)		Lower TsEn in AD group than the normal
